# Effectiveness of Physical Rehabilitation Interventions on Walking Capacity and Wearable Sensor—Derived Performance After Stroke: A Systematic Review and Meta-Analysis of Randomized Controlled Trials

**DOI:** 10.3390/s26144332

**Published:** 2026-07-08

**Authors:** Joy C. Ezeugwa, Aiza Khan, Deborah O. Okusanya, Liz Dennett, Brian H. Buck, Patricia J. Manns, Victor E. Ezeugwu

**Affiliations:** 1 Faculty of Rehabilitation Medicine, University of Alberta, Edmonton, AB T6G 2G4, Canada; 2 Geoffrey and Robyn Sperber Health Sciences Library, University of Alberta, Edmonton, AB T6G 1H9, Canada; 3 Division of Neurology, Department of Medicine, University of Alberta, Edmonton, AB T6G 2B7, Canada

**Keywords:** stroke, walking, rehabilitation, wearable sensors, precision health, meta-analysis

## Abstract

**Highlights:**

**What are the main findings?**

- Exercise interventions significantly improve functional walking capacity (gait speed and endurance) but yield minimal changes in real-world daily step counts.
- Behaviour change techniques, either alone or combined with exercise, are associated with larger magnitude of effects in real-world walking outcomes.

**What are the implications of the main findings?**

- After stroke, what people are capable of doing in a controlled setting does not always match what they actually do in everyday life.
- Continuous, unsupervised, wearable sensor-derived performance data offer insights not captured by traditional capacity-focused assessments.

**Abstract:**

Improvements in walking capacity after stroke do not always translate into increased real-world performance, highlighting a critical gap in promoting long-term health after rehabilitation. This systematic review and meta-analysis evaluated the effects of physical rehabilitation interventions, including exercise, behaviour change techniques (BCTs), and combined approaches, on wearable sensor-derived movement behaviours and walking capacity in people with stroke. Four databases (MEDLINE, Embase, CINAHL, and Scopus) were searched through May 2026 for randomized controlled trials comparing these interventions with usual care or other interventions. Outcomes included steps per day, gait speed, and walking endurance (6-min walk test; 6MWT). Random-effects meta-analyses were conducted, reporting standardized mean differences (SMDs) and 95% confidence intervals (CIs). Thirty-one studies met inclusion criteria, and 26 distinct studies were included in the meta-analyses. For real-world performance, BCT-only interventions demonstrated the largest pooled effect estimate on steps per day (SMD = 0.45; 95% CI: 0.22–0.69), followed by combined BCT + Exercise interventions (SMD = 0.38; 95% CI: 0.10–0.66), while Exercise-only interventions showed a smaller but statistically significant effect (SMD = 0.23; 95% CI: 0.02–0.44). Conversely, Exercise-only interventions were the only category associated with statistically significant improvements in walking capacity, improving both self-selected gait speed (SMD = 0.36; 95% CI: 0.13–0.58) and walking endurance (6MWT; SMD = 0.41; 95% CI: 0.25–0.57). In contrast, neither combined BCT + Exercise nor BCT-only interventions demonstrated statistically significant effects across gait speed or endurance outcomes. These findings suggest that exercise improves walking capacity, whereas interventions incorporating BCTs may have greater effects on real-world walking performance after stroke.

## 1. Introduction

Stroke remains a leading global health challenge, affecting over 25 million individuals worldwide [1]. Many stroke survivors experience persistent impairments that limit mobility and reduce their ability to engage in rehabilitation and achieve recommended levels of physical activity (PA) [2,3]. Stroke recurrence is common, with nearly one in four stroke survivors experiencing a subsequent event after five years [4], underscoring the importance of addressing modifiable risk factors such as physical inactivity and prolonged sedentary behaviour (SB, defined as sitting or lying activities requiring ≤1.5 metabolic equivalent tasks (METs) during waking hours) [5]. Both physical inactivity and prolonged SB are associated with increased cardiovascular risk and may contribute to stroke recurrence and mortality [6,7,8,9,10]. Guidelines recommend 20 to 60 min of aerobic exercise three to five days per week, in addition to strength and flexibility training [2,11]. Despite these recommendations, stroke survivors remain largely inactive and accumulate substantial sedentary time [3,12,13]. 

Walking is a simple, accessible, and commonly prescribed form of PA after stroke, with daily step count associated with improved cardiovascular and metabolic outcomes [14,15,16]. Walking outcomes can be broadly categorized into *capacity* (e.g., gait speed, walking endurance), representing effort under standardized conditions, and *performance* (e.g., daily steps), reflecting real-world activity [17]. While rehabilitation programmes typically target improvements in walking capacity through structured training [18,19,20], growing evidence suggests that gains in capacity do not consistently correspond with improvements in real-world performance [14,21,22,23]. This capacity–performance gap represents a key challenge in post-stroke rehabilitation.

Behavioural interventions, particularly those incorporating behaviour change techniques (BCTs), such as goal setting, self-monitoring, and feedback, have been proposed to address this gap [24,25,26,27]. These approaches may support sustained increases in daily activity, particularly following structured rehabilitation and discharge into the community [28,29,30], which is relevant for long-term cardiovascular health and secondary stroke prevention. However, evidence regarding the relative effectiveness of Exercise-only, BCT-only, and combined interventions on both walking capacity and real-world performance remains unclear. Although several systematic reviews have examined PA interventions after stroke [31,32,33,34,35,36,37,38], few have simultaneously evaluated both capacity and performance outcomes, and to our knowledge, none have quantitatively compared intervention types across these domains using wearable sensor-derived measures of daily performance. Therefore, this systematic review and meta-analysis aimed to consolidate evidence from randomized controlled trials (RCTs) to evaluate the effectiveness of physical rehabilitation interventions, including Exercise-only, BCT-only, and combined approaches, on walking capacity and wearable sensor-derived performance outcomes after stroke. Our results may strengthen the evidence base for identifying interventions that improve both capacity and real-world performance and inform the development of targeted rehabilitation strategies.

## 2. Materials and Methods

### 2.1. Design

This systematic review and meta-analysis of RCTs was conducted in accordance with the Preferred Reporting Items for Systematic reviews and Meta-Analyses (PRISMA) guidelines (Supplementary Table S1). The study protocol was prospectively registered with PROSPERO (CRD42023411679).

### 2.2. Deviations from Protocol

Minor deviations from the registered protocol were made during the execution of this review. First, the title was updated to more accurately reflect the final, precise scope of the synthesized evidence base. Second, while the Timed Up and Go (TUG) test and the modified Rankin Scale (mRS) were initially listed as secondary outcomes during the registration phase, they were subsequently excluded from the final analysis as they do not measure walking capacity or daily performance. All other outcomes were retained and analyzed as planned.

### 2.3. Literature Search and Databases

Searches were conducted in MEDLINE (Ovid), Embase (Ovid), CINAHL (EBSCOhost), and Scopus. Searches were initially conducted on 7 March 2023 and updated on 25 May 2026, with no restrictions on publication date. MEDLINE via Ovid is a curated, indexed core database, which is recommended for comprehensive, structured, or systematic searches that capture Medical Subject Headings (MeSH) and keywords. Database-specific records identified were: MEDLINE (*n* = 471), Embase (*n* = 695), CINAHL (*n* = 215), and Scopus (*n* = 773), yielding 2154 records from database searches and an additional 18 records from citation searching of other sources. While Scopus does not utilize MeSH terms, it was intentionally included to capture interdisciplinary records from engineering, physical and social sciences. Search strategies combined terms related to wearable sensors, stroke, and movement behaviours, with filters for RCTs, and were adapted for each database. Records were imported into Covidence (Veritas Health Innovation, Melbourne, Australia) for deduplication. Full search strategies are provided in Supplementary Table S2.

### 2.4. Study Selection and Eligibility Criteria

Three independent reviewers (JCE, AK, and DOO) screened titles, abstracts, and full-text articles for eligibility, with each record undergoing dual independent evaluation. Disagreements were resolved through adjudication by a senior author (VEE). Inter-rater agreement was high (proportionate agreement 0.90–0.96; Cohen’s kappa = 0.50).

#### PICO Criteria

Eligibility criteria were defined a priori based on the population, intervention, comparator, and outcome (PICO) framework. The review included randomized controlled trials (RCTs) evaluating adults aged 18 years or older diagnosed with stroke (population). Eligible interventions comprised physical rehabilitation approaches categorized as Exercise-only, BCTs-only, or combined Exercise and BCT approaches (intervention). Interventions based exclusively on exoskeletons or robotics were excluded, as these are not commonly part of routine physical rehabilitation. These programmes were compared against usual care or active controls involving a different intensity or type of physical rehabilitation (comparator). All included studies were required to use wearable sensors to objectively capture habitual movement metrics. Primary outcomes of interest included measures of objective real-world walking performance derived explicitly from wearable sensor-derived daily activity behaviours (such as daily step counts), as well as laboratory- or clinic-based functional walking capacity measures, specifically self-selected and fastest gait speed and walking endurance measured via the 6-min walk test (outcomes). Studies were excluded if outcomes were limited to upper extremity function, if participants were aged under 18 years, or if they utilized non-randomized designs (e.g., observational, cohort, or cross-sectional studies). Finally, systematic reviews, meta-analyses, and protocol papers without published results were excluded from synthesis.

### 2.5. Data Extraction

Data were extracted independently by two authors (JCE and DOO) using a standardized form (Supplementary Table S3). Extracted data included study characteristics (e.g., authors, year, sample size at baseline and follow-up, setting), participant characteristics (e.g., age, sex, time since stroke, stroke type and severity), and intervention and control details, including type (Exercise, BCTs, or combined) and components based on frequency, intensity, time, type, and setting (FITTS), as well as information on wearable sensors (type and placement). Outcomes were categorized as walking performance or walking capacity. Outcome data were extracted at the end of the intervention or follow-up; for studies with multiple time points, end-of-intervention data were used. Effect estimates, adherence, and adverse events were extracted where reported, with efforts made to contact study authors for missing data. Studies were selected for final meta-analysis if they provided sufficient quantitative data (sample sizes, post-intervention means, and standard deviations or standard errors) for at least one primary performance or capacity outcome. For multi-arm trials contributing more than one comparison to a pooled analysis, shared control groups were divided across comparisons in accordance with Cochrane recommendations to avoid double-counting participants.

### 2.6. Assessment of Risk of Bias and Quality of Evidence

Risk of bias (internal validity and statistical quality) was assessed independently by two authors (JCE and VEE) using the Physiotherapy Evidence Database (PEDro) on a 0–10 scale [39], and disagreements were resolved by consensus. PEDro scores were interpreted using established thresholds [39]. The certainty of evidence for each pooled outcome was evaluated using the Grading of Recommendations Assessment, Development and Evaluation (GRADE) approach [40], considering risk of bias, inconsistency, indirectness, imprecision, and publication bias. Certainty was classified as high (very confident the estimate is close to the true effect), moderate (likely close, but may differ), low (may differ substantially), or very low (likely to differ substantially) [40].

### 2.7. Data Analysis

Study-level outcome data were extracted and synthesized narratively. Meta-analyses were conducted for outcomes reported in at least two studies to estimate between-group intervention effects. Pooled estimates were calculated using the generic inverse variance method under a random-effects model (DerSimonian–Laird estimator) to account for between-study heterogeneity. Effect sizes were expressed as standardized mean differences (SMDs) with 95% confidence intervals (CIs) for daily steps, gait speed (self-selected and fastest), and walking endurance. Statistical heterogeneity was assessed using the I-squared statistic [41], with values of <25%, 25–50%, and >50% representing low, moderate, and substantial heterogeneity, respectively [41]. Studies reporting medians and interquartile ranges were excluded from meta-analysis but included in the descriptive synthesis. Where studies included multiple eligible intervention arms, we followed Cochrane recommendations to maintain subgroup fidelity and preserve the independence of distinct treatment categories. Each unique intervention arm was entered as a separate comparison entry within its respective subgroup meta-analysis pool, allowing us to isolate specific treatment effects against the shared control group. Effect sizes were interpreted using thresholds proposed for rehabilitation research: small (0.08–0.15), moderate (0.19–0.36), and large (0.41–0.67) [42]. Results are presented using forest plots and summary tables. Sensitivity analyses were conducted using subgroup analyses stratified by intervention type. All analyses were done using STATA 18 (StataCorp, College Station, TX, USA) and “metafor” package in R version 5.0-1 (Auckland, New Zealand).

## 3. Results

### 3.1. Study Selection

**Figure 1:**
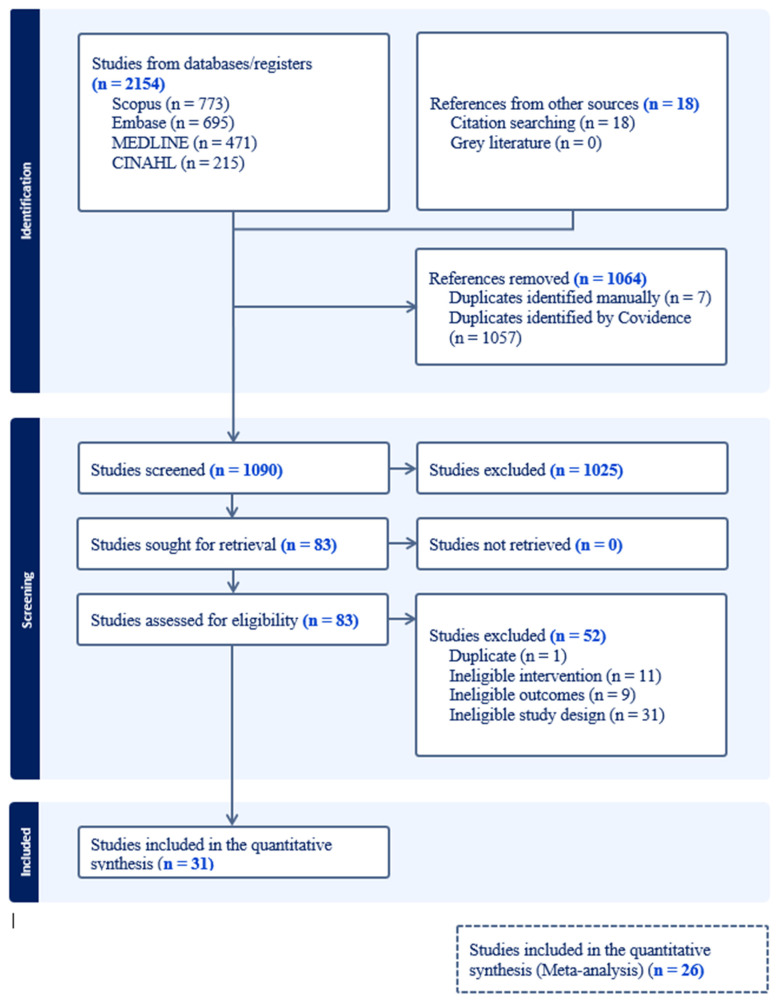
PRISMA 2020 flow diagram.

A total of 2154 records were identified (2154 from databases and 18 through citation searching). After removal of duplicates, 1090 records were screened, and 83 full-text articles were assessed for eligibility. Of these, 52 were excluded due to study design (*n* = 31), intervention (*n* = 11), outcome (*n* = 9), or duplication identified at full-text review (*n* = 1). A total of 31 studies were included in the qualitative synthesis [14,22,29,43,44,45,46,47,48,49,50,51,52,53,54,55,56,57,58,59,60,61,62,63,64,65,66,67,68,69,70], of which 26 studies were included in the meta-analysis [14,22,29,43,44,45,46,48,50,51,52,53,54,55,56,57,58,59,60,61,63,65,66,68,69,70]. The flow chart for study selection is shown in Figure 1.

### 3.2. Study Characteristics

The included studies were published between 2009 and 2026. Geographically, eight studies were conducted in the United States [14,46,47,48,61,63,64,67], four in Japan [43,44,50,52], four in Australia [45,49,60,70], two each in Canada [51,55], France [53,58], two in the United Kingdom [66,68]. One study each was conducted in Belgium [62], Brazil [69], Denmark [54], India [29], Israel [65], New Zealand [22], the Netherlands [56], Norway [57], and Singapore [59]. A total of 2520 participants were included, with 1385 in intervention groups and 1135 in control groups. Of these, 937 were female (533 in intervention groups and 404 in control groups). Study characteristics are summarized in Table 1.

The mean age ranged from 48 to 73.0 years in the intervention groups and from 48 to 71.8 years in the control groups. Seventeen studies [43,44,45,47,48,50,51,52,53,54,55,56,57,58,61,62,69] included participants in the acute or subacute phase, while 14 studies [14,22,29,46,49,59,60,63,64,65,66,67,68,70] included participants in the chronic phase. Time since stroke was reported in 24 studies and ranged from 0.12 to 74.4 months in the intervention groups and from 0.12 to 96.9 months in the control groups. Ischemic stroke was the most prevalent type, comprising 57.1% to 100% of participants in the intervention groups and 54.2% to 100% in the control groups. Stroke severity was most commonly assessed using the National Institutes of Health Stroke Scale (NIHSS) and ranged from 0.9 to 6 in the intervention groups and from 0.8 to 6 in the control groups.

### 3.3. Interventions Characteristics

Table 2 presents the intervention characteristics and wearable sensors used for activity monitoring. Of the 31 included studies, 26 [14,22,29,43,44,45,46,48,49,50,52,54,55,57,59,60,61,62,63,64,65,66,67,68,69,70] reported performance outcomes, primarily steps per day, and 20 studies [14,29,43,44,45,46,48,49,50,51,53,55,56,57,58,63,64,65,68,69] reported capacity outcomes, including gait speed and/or 6MWT. Interventions were grouped into three categories: BCT-only, Exercise-only, or combined. Eight studies included BCT-only interventions, commonly involving goal setting, self-monitoring, feedback, education, or gamification to increase activity or reduce sedentary time [43,44,47,49,53,58,67,69]. Fourteen studies evaluated Exercise-only interventions, including treadmill training, high-intensity interval training, circuit-based exercise, task-specific training, gait training, strengthening, and home- or community-based exercise programmes [22,48,51,54,56,57,59,60,61,63,64,65,66,68]. Nine studies included combined Exercise and BCT interventions, integrating structured exercise with strategies such as goal setting, feedback, self-monitoring, counselling, and education [14,29,45,46,50,52,55,62,70]. Of note, four studies included multiple intervention arms [14,48,51,63]. Detailed BCT classifications, coded using the BCT Taxonomy, are provided in Supplementary Table S4.

### 3.4. Characteristics of Activity Monitors and Wearable Sensor Protocols

The included studies demonstrated substantial variation in wearable sensor characteristics, including device grade (research versus consumer), anatomical placement, and monitoring protocols (Table 2). Research-grade devices accounted for the majority of sensors used and included the Step Activity Monitor (SAM), ActiGraph, activPAL sensors. The SAM, worn exclusively at the ankle, was the most frequently used device (*n* = 7 studies) [14,22,46,48,63,64,66]. This was followed by various models of the ActiGraph sensor (*n* = 5 studies) [29,49,57,61,69], which were typically worn on the waist [49,57,61,69] and, in one study, on the unaffected hip [29]. Thigh-worn activPAL sensors, which incorporate both inclinometer and accelerometer functions, were used in three studies [45,49,68].

Fitbit was the most commonly used consumer-grade device, reported in five studies [14,50,51,67,70]. Fitbit sensors were worn at the ankle in three studies [14,50,51] and at the wrist in two studies [67,70]. Across studies, ankle-mounted sensors were the most common placement, and monitoring protocols generally involved at least three full days of wear.

### 3.5. Methodological Quality

Methodological quality, assessed using the PEDro scale, ranged from 5 to 8 out of 10 (Supplementary Table S5), indicating overall moderate to good quality across included studies. Most studies demonstrated strong internal validity, with all reporting random allocation, baseline comparability, and between-group statistical comparisons. Blinding of participants and therapists was not achieved in any of the included studies, which is expected in rehabilitation trials. Assessor blinding was reported in approximately half of the studies. The majority of studies reported low attrition (<15%) and provided point estimates with measures of variability. Intention-to-treat analyses were less consistently applied. Overall, the included trials were of acceptable methodological quality, with common limitations related to blinding.

The GRADE certainty of evidence was moderate for Exercise-only and BCT-only interventions on daily steps, gait speed, and walking endurance, but very low for most combined interventions and fastest gait speed outcomes. Downgrading was primarily driven by imprecision and inconsistency (Supplementary Table S6).

### 3.6. Meta-Analysis of Intervention Effects

The pooled intervention effects are summarized in Table 3. BCT-only interventions were associated with the largest pooled effect estimate for steps per day, with no observed statistical heterogeneity, followed by combined BCT + Exercise interventions, whereas Exercise-only interventions demonstrated the smallest effect. For walking capacity outcomes, Exercise-only interventions were the only category associated with statistically significant improvements, specifically in self-selected gait speed and 6-min walk test performance. In contrast, neither combined BCT + Exercise nor BCT-only interventions demonstrated statistically significant effects on gait speed or walking endurance outcomes.

#### 3.6.1. Performance Outcome

##### Steps per Day

**Figure 2:**
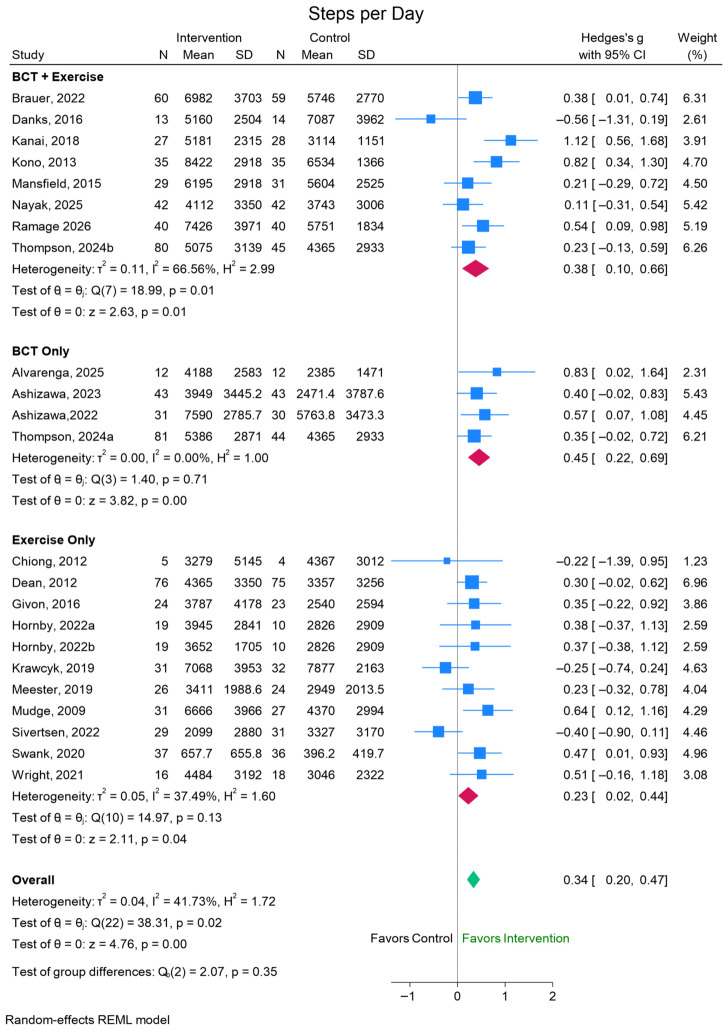
Forest plot of the effectiveness of combined BCT + Exercise, BCT-only, and Exercise-only interventions on steps per day [14,22,29,43,44,45,46,50,52,54,55,57,59,60,61,63,65,66,68,69,70].

A total of 21 unique studies comprising 23 analysis entries and 1628 participants contributed to the steps-per-day meta-analysis (Figure 2). Overall, interventions increased daily steps compared with control conditions (Hedges’s g = 0.34, 95% CI [0.20, 0.47], z = 4.76, *p* < 0.001), with moderate heterogeneity across studies (I^2^ = 41.7%, *p* = 0.02). Interventions incorporating BCTs demonstrated the largest pooled effect on steps per day (Hedges’s g = 0.45, 95% CI [0.22, 0.69]; four entries), with no observed heterogeneity (I^2^ = 0.0%). Combined BCT + Exercise interventions also demonstrated a moderate effect (Hedges’s g = 0.38, 95% CI [0.10, 0.66]; eight entries), although heterogeneity was substantial (I^2^ = 66.6%). Exercise-only interventions showed a smaller but statistically significant effect on steps per day (Hedges’s g = 0.23, 95% CI [0.02, 0.44]; 11 entries), with low-to-moderate heterogeneity (I^2^ = 37.5%). However, the formal test for subgroup differences was not statistically significant (Qb(2) = 2.07, *p* = 0.35), indicating no statistically significant differences in effect size between intervention categories.

#### 3.6.2. Capacity Outcomes

##### Self-Selected Gait Speed

**Figure 3:**
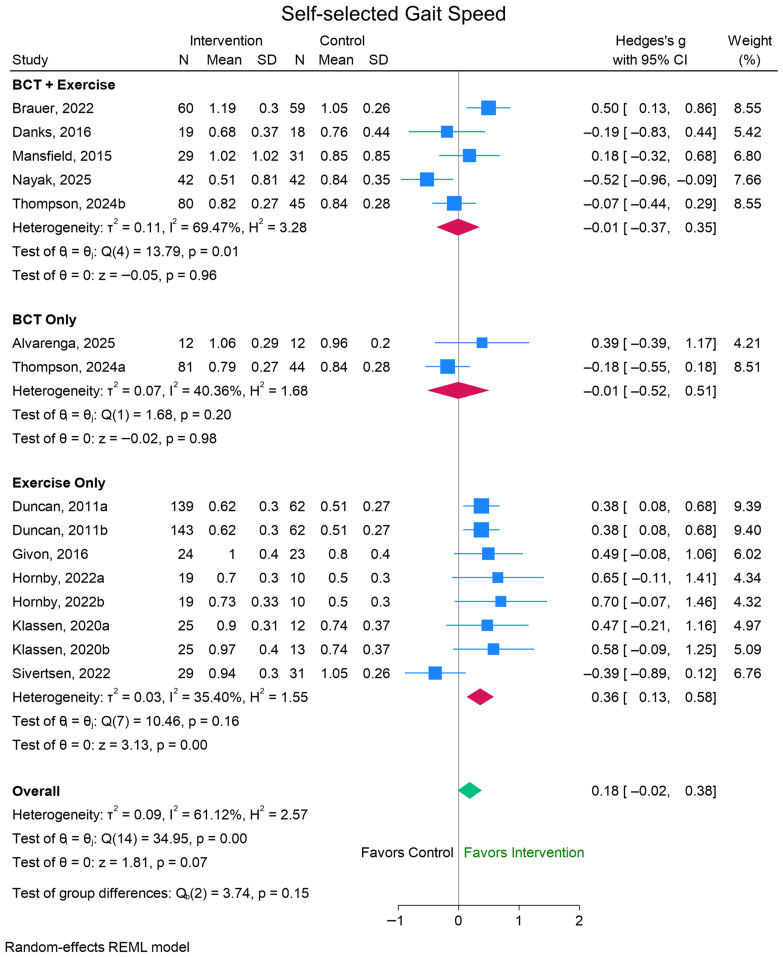
Forest plot of the effectiveness of combined BCT + Exercise, BCT-only, and Exercise-only interventions on self-selected gait speed [14,29,45,46,48,51,55,57,63,65,69].

A total of 11 unique studies comprising 15 analysis entries and 1482 participants evaluated self-selected gait speed (Figure 3). Overall, interventions demonstrated a small, non-significant improvement in self-selected gait speed compared with control conditions (Hedges’s g = 0.18, 95% CI [−0.02, 0.38], z = 1.81, *p* = 0.07), with substantial heterogeneity across studies (I^2^ = 61.1%, *p* < 0.001).

Exercise-only interventions demonstrated the largest pooled effect on self-selected gait speed (Hedges’s g = 0.36, 95% CI [0.13, 0.58]; eight entries), with low-to-moderate heterogeneity (I^2^ = 35.4%). In contrast, pooled effects for BCT + Exercise interventions (Hedges’s g = −0.01, 95% CI [−0.37, 0.35]; five entries) and BCT-only interventions (Hedges’s g = −0.01, 95% CI [−0.52, 0.51]; two entries) indicated little to no effect on self-selected gait speed. Heterogeneity was substantial for BCT + Exercise interventions (I^2^ = 69.5%) and moderate for BCT-only interventions (I^2^ = 40.4%).

However, the formal test for subgroup differences was not statistically significant (Qb(2) = 3.74, *p* = 0.15), indicating no statistically significant differences in effect size between intervention categories.

##### Fastest Gait Speed

**Figure 4:**
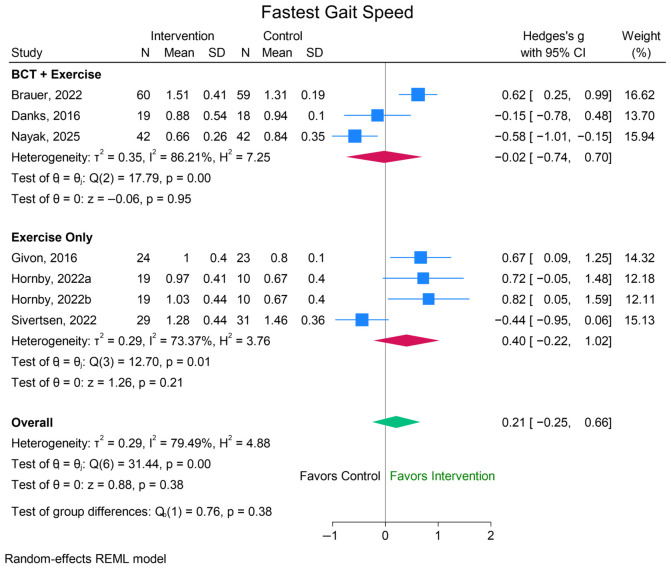
Forest plot of the effectiveness of combined BCT + Exercise, BCT-only, and Exercise-only interventions on fastest gait speed [29,45,46,57,63,65].

A total of six unique studies comprising seven analysis entries and 425 participants evaluated fastest gait speed (Figure 4). Overall, interventions did not significantly improve fastest gait speed compared with control conditions (Hedges’s g = 0.21, 95% CI [−0.25, 0.66], z = 0.88, *p* = 0.38), with substantial heterogeneity across studies (I^2^ = 79.5%, *p* < 0.001). Exercise-only interventions demonstrated the largest pooled effect estimate on fastest gait speed (Hedges’s g = 0.40, 95% CI [−0.22, 1.02]; four entries), although the confidence interval included no effect and heterogeneity was substantial (I^2^ = 73.4%). Similarly, combined BCT + Exercise interventions demonstrated little to no overall effect on fastest gait speed (Hedges’s g = −0.02, 95% CI [−0.74, 0.70]; 3 entries), with substantial heterogeneity (I^2^ = 86.2%). No pooled analysis was available for BCT-only interventions. The formal test for subgroup differences was not statistically significant (Qb(1) = 0.76, *p* = 0.38), indicating no statistically significant differences in effect size between intervention categories.

##### Walking Endurance (6-Min Walk Test)

**Figure 5:**
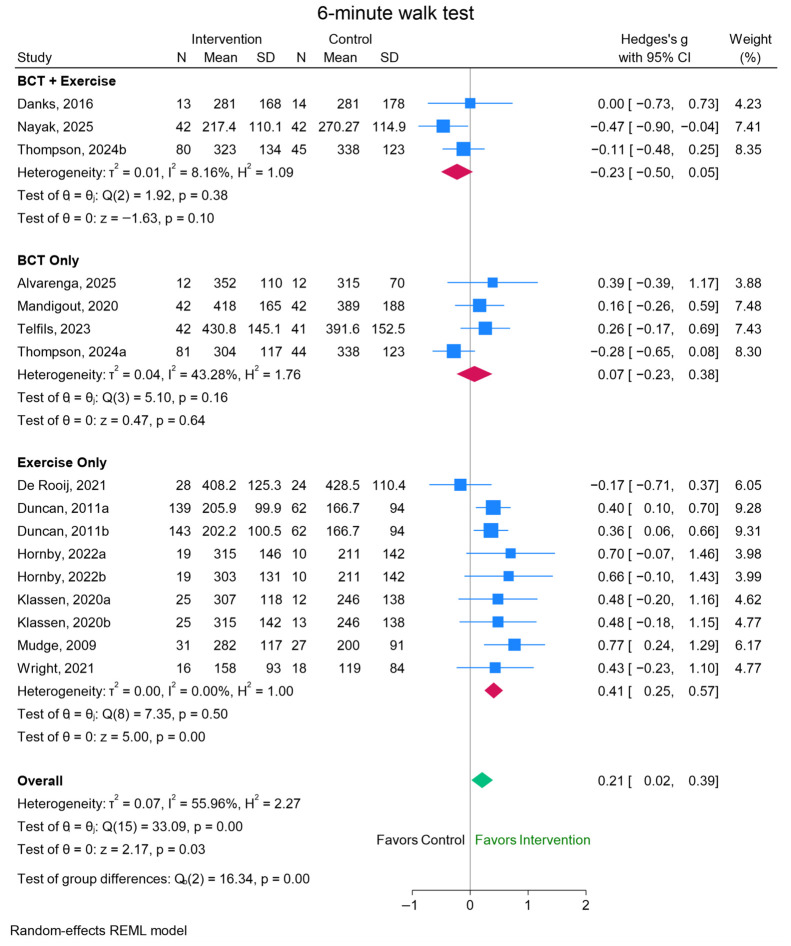
Forest plot of the effectiveness of combined BCT + Exercise, BCT-only, and Exercise-only interventions on 6-min walk test [14,22,29,46,48,51,53,56,63,68,69].

A total of 12 unique studies comprising 16 analysis entries and 1502 participants evaluated walking endurance, as measured by the 6-min walk test (Figure 5). Overall, interventions demonstrated a small but statistically significant improvement in walking endurance compared with control conditions (Hedges’s g = 0.21, 95% CI [0.02, 0.39], z = 2.17, *p* = 0.03), with moderate heterogeneity across studies (I^2^ = 56.0%, *p* < 0.001). Exercise-only interventions demonstrated the largest pooled effect on walking endurance and were the only subgroup associated with statistically significant improvements (Hedges’s g = 0.41, 95% CI [0.25, 0.57]; nine entries), with no observed heterogeneity (I^2^ = 0.0%). In contrast, BCT-only interventions demonstrated little overall effect on walking endurance (Hedges’s g = 0.07, 95% CI [−0.23, 0.38]; four entries), with moderate heterogeneity (I^2^ = 43.3%). Combined BCT + Exercise interventions demonstrated a small negative, non-significant pooled effect estimate (Hedges’s g = −0.23, 95% CI [−0.50, 0.05]; three entries), with low heterogeneity (I^2^ = 8.2%). A formal test for subgroup differences was statistically significant (Qb(2) = 16.34, *p* < 0.001), indicating that intervention effects on walking endurance differed between intervention categories. Exercise-only interventions demonstrated significantly larger effect estimates than BCT-only and combined BCT + Exercise interventions.

## 4. Discussion

Current post-stroke rehabilitation paradigms often prioritize capacity-based outcomes (e.g., gait speed, 6MWT) over real-world performance (e.g., daily steps). However, growing evidence suggests that improvements in capacity do not consistently translate into increased daily physical activity (PA) [14,21,22,23,25]. This disconnect between what individuals can do in clinical settings and what they actually do in daily life represents a key gap in post-stroke rehabilitation. This capacity–performance gap is increasingly recognized as a major challenge in stroke rehabilitation and may help explain why improvements observed in clinical settings often fail to produce meaningful changes in community participation [14,23]. Our systematic review and meta-analysis addressed this issue by evaluating the effects of Exercise-only, BCT-only, and combined Exercise + BCT interventions on both objectively measured performance and capacity outcomes after stroke.

Our findings suggest distinct patterns across performance and capacity outcomes. For daily performance (step counts), BCT-only interventions showed the largest pooled effect estimate with no observed heterogeneity, followed by combined BCT + Exercise interventions, while Exercise-only interventions showed the smallest effect. Conversely, Exercise-only interventions consistently demonstrated the largest effects across walking capacity outcomes. Exercise-only interventions were the only category associated with significant improvements in self-selected gait speed and walking endurance, whereas BCT-only and combined BCT + Exercise interventions demonstrated little to no overall effect on these outcomes. Collectively, these findings suggest that BCT-focused interventions may be particularly useful for increasing real-world performance, whereas Exercise-based interventions appear fundamental for improving walking capacity after stroke.

The finding that BCT-only interventions showed the largest pooled effect estimate for steps per day, supported by moderate-certainty evidence, is consistent with previous evidence demonstrating that behavioural strategies such as goal setting, self-monitoring, feedback, and action planning can effectively increase physical activity participation among individuals with chronic health conditions, including stroke [14,35,71,72]. Such strategies directly target behavioural determinants of activity engagement and may help individuals improve self-efficacy and overcome performance barriers in everyday activities [28,73,74,75]. On the other hand, Exercise-only interventions were the only category associated with significant improvements in self-selected gait speed and walking endurance, supported by moderate-certainty evidence. These results align with established principles of overload, specificity, and task-oriented training, whereby physiological adaptations require sufficient intensity, repetition, and practice volume [11,19]. Unlike behaviourally focused approaches, exercise interventions directly target the neuromuscular, cardiovascular, and biomechanical mechanisms underlying walking performance, thereby providing the stimulus necessary to improve physical capacity.

A notable finding was the underperformance of combined Exercise + BCT interventions across outcomes, remaining non-significant for self-selected gait speed, fastest gait speed, and walking endurance. Although conceptually appealing as dual-target interventions, combined approaches did not consistently outperform single-component interventions. Several explanations are possible, including reduced exercise dose, competing intervention priorities, insufficient fidelity of behavioural delivery, or heterogeneity in intervention design and implementation. However, these hypotheses remain speculative because intervention fidelity and adherence were inconsistently reported. Overall, these findings suggest that increasing intervention complexity does not necessarily result in superior outcomes and that intervention components should be aligned with the outcome being targeted. Exercise-based interventions appear best suited for improving capacity (e.g., gait speed and endurance), whereas BCT-based interventions are particularly effective for enhancing real-world performance (e.g., daily step counts).

Interpretation of the findings should also consider the wearable sensor technologies used across studies. Variability in device type, anatomical placement, and proprietary processing algorithms may have contributed to heterogeneity in step-count outcomes. In people with limited mobility such as after stroke, ankle- and thigh-worn sensors generally demonstrate greater sensitivity for detecting slow and asymmetric gait patterns, whereas wrist-worn devices may be more susceptible to measurement error [76,77,78,79]. These findings highlight the importance of considering wearable sensor characteristics when interpreting performance-based outcomes in stroke rehabilitation research.

### 4.1. Limitations

This review has several limitations. First, although the search strategy was comprehensive, relevant studies may have been missed due to variability in terminology or incomplete reporting of intervention content. Second, substantial heterogeneity was observed across studies in terms of design, participant characteristics (e.g., stroke chronicity), intervention intensity, and outcome measures, particularly for combined interventions. Third, classification of BCTs relied on published descriptions, which may not fully reflect fidelity or quality of delivery. Fourth, publication bias cannot be excluded, and the number of studies in certain subgroups (e.g., BCT-only for capacity outcomes) was limited, reducing statistical power. Finally, this review focused on quantitative outcomes; qualitative aspects such as feasibility, acceptability, and patient experience were not examined.

### 4.2. Clinical Implications and Future Directions

For clinicians and rehabilitation teams, these findings emphasize the importance of tailoring interventions according to rehabilitation goals [80,81]. When the primary objective is to increase real-world physical activity, behavioural strategies such as goal setting, self-monitoring, feedback, and action planning may be particularly useful for supporting increases in daily physical performance. Conversely, when the goal is to improve walking capacity, especially gait speed and endurance, structured exercise interventions should remain a central component of rehabilitation programming [19]. The differing effects observed across performance and capacity outcomes also highlight the ongoing challenge of translating gains achieved in rehabilitation into sustained changes in everyday behaviour. Future studies should investigate whether sequential intervention models—where exercise is first used to improve walking capacity and behavioural strategies are subsequently introduced to support long-term activity participation—can more effectively bridge this capacity–performance gap following stroke.

## 5. Conclusions

This systematic review and meta-analysis demonstrated distinct patterns across performance and capacity outcomes following stroke. Intervention categories incorporating BCTs demonstrated numerically larger pooled effects for daily step counts, whereas Exercise-only interventions consistently demonstrated the largest effects on walking capacity and were the only category associated with significant improvements in self-selected gait speed and walking endurance. The strongest evidence for differential effects was observed for walking endurance, where intervention category significantly influenced outcomes. These findings highlight the importance of aligning intervention selection with rehabilitation goals. BCT-focused interventions may be particularly useful for increasing real-world physical activity, whereas Exercise-only interventions appear fundamental for improving walking capacity.

## Figures and Tables

**Table 1:** Characteristics of included studies.

Study (Author, Year, Country)	n (I, C)	Age (Years) (I, C)	Female (%) (I, C)	Time Since Stroke (Months) (I, C)	Ischemic Stroke (%) (I, C)	Stroke Severity (I, C)	Intervention Type	Setting
Alvarenga et al. (2022), Brazil [69]	12, 12	70 ± 11, 64 ± 11	58.0, 58.0	2.0 ± 1.0 3.0 ± 1.0	75, 92	NR	BCT	Home
Ashizawa et al. (2022), Japan [43]	31, 30	72.3 ± 8.9, 70.3 ± 7.7	38.7, 30.0	NR	100, 100	NIHSS: 1.2 ± 1.4, 0.8 ± 1.0	BCT	Hospital/Home
Ashizawa et al. (2023), Japan [44]	43, 43	72.0 ± 8.4, 71.8 ± 7.6	32.6, 32.6	NR	100, 100	NIHSS: 1.0 ± 1.3, 0.9 ± 1.0	BCT	Hospital/Home
Brauer et al. (2022), Australia [45]	60, 59	62.0 ± 11.0, 64.0 ± 9.0	20.0, 22.0	0.9 ± 0.5	78.0, 86.0	mRS: 2.8 ± 0.6, 2.9 ± 0.6	Combined	Inpatient Rehabilitation
Chiong et al. (2012), Singapore [59]	5, 4	48 ± 21, 48 ± 17	40, 50	19.0 (20.5), 24.5 (18.8)	80, 75	MAS: 1.0 (1.0), 0 (0.8)	Combined	Outpatient Rehabilitation
Danks et al. (2016), United States [46]	13, 14	59.1 ± 8.7, 58.2 ± 12.4	46.2, 43.0	29.4 ± 21.4, 50.8 ± 44.1	NR	FMA-LE: 16.8 ± 7.1, 18.6 ± 4.6	Combined	Laboratory
Dean et al. (2012), Australia [60]	76, 75	66.7 ± 14.3, 67.5 ± 10.2	50.0, 46.7	80.4 ± 80.4, 62.4 ± 64.8	NR	NR	Exercise	Home and Community
De Rooij et al. (2021), Netherlands [56]	28, 24	65 [57–70], 61 [53–71]	35.7, 25.0	2.8 [2.3–3.7], 2.2 [1.7–3.4]	85.7, 83.3	FAC ≥ 5: 82.1, 62.5%	Exercise	Outpatient Rehabilitation
Duncan et al. (2011), United States [48] *	139/143, 126	60.1 ± 12.3, 63.3 ± 12.5	38.8/48.3, 48.4	2.1 ± 0.3/ 2.1 ± 0.3, 2.1 ± 0.3	68.4/73.5, 71.4	mRS 3–4: 91.4/83.9, 83.3%	Exercise	Inpatient Rehabilitation and Home
English et al. (2016), Australia [49]	19, 14	65.4 ± 12.3, 67.8 ± 13.8	31.6, 35.7	33.6 ± 31.2, 49.2 ± 51.6	89.5, 57.1	NIHSS > 4: 26.3%, 50.0%	BCT	Home and Community
Givon et al. (2016), Israel [65]	24, 23	56.7 ± 9.3, 62.0 ± 9.3	54.2, 26.1	36.0 ± 21.6, 31.2 ± 21.6	87.5, 82.6	FMA-UE: 32.2 ± 20.5, 26.5 ± 19.6	Exercise	Community
Hornby et al. (2022), United States [63] *	19/19, 20	56 ± 11/ 60 ± 9.3, 54 ± 13	15.8/42.1, 50.0	NR	NR	FMA-LE: 23 ± 5.7/24 ± 4.5, 21 ± 5.1	Exercise	Laboratory
Ivey et al. (2015), United States [64]	18, 16	61, 63	44.4, 31.3	NR	NR	NR	Exercise	Laboratory
Kanai et al. (2018), Japan [50]	23, 25	66.8 ± 10, 62.9 ± 9.1	34.8, 48.0	0.12 ± 0.05, 0.13 ± 0.05	100, 100	NIHSS: 0.9 ± 0.8, 1.0 ± 1.0	Combined	Hospital
Klassen et al. (2020), Canada [51] *	25/25, 24	56.0 ± 11.0/ 58.0 ± 10.0, 58.0 ± 13.0	36.0/44.0, 42	0.9 ± 0.3/ 1.0 ± 0.3, 0.9 ± 0.4	88/76, 83.5	NIHSS: 5 ± 3/5 ± 3, 5 ± 3	Exercise	Inpatient Rehabilitation
Kono et al. (2013), Japan [52]	35, 35	63.5 ± 7.0, 63.4 ± 11.4	40.0, 22.9	NR	100, 100	Both-NIHSS: 2, 2	Combined	Home
Krawcyk et al. (2019) Denmark [54]	31, 32	63.7 ± 8.9, 63.4 ± 11.4	26.0, 19.0	<1 month	100, 100	SSS: 54.6 ± 5.8, 55.3 ± 4.4	Exercise	Home
Mandigout et al. (2020), France [53]	41, 42	63.0 ± 12.0, 58.0 ± 24.0	26.8, 23.8	2.3 ± 1.6, 2.4 ± 1.6	75.6, 73.8	MI: 96 [19], 92.5 [27]	BCT	Home
Mansfield et al. (2015), Canada [55]	29, 28	64.0 [19], 61.5 [13]	31.0, 43.0	0.9 ± 0.7, 0.8 ± 0.7	83, 79	NIHSS: 2 [2], 1 [3]	Combined	Inpatient Rehabilitation
Meester et al. (2019), United Kingdom [66]	26, 24	60.9 ± 14.9, 62.3 ± 15.3	42.3, 54.2	60.2 ± 62.2, 25.7 ± 32.7	69.2, 54.2	NR	Exercise	Community
Mudge et al. (2009), New Zealand [22]	31, 27	76.0 [39.0–89.0], 71.0 [44.0–86.0]	38.7, 51.9	69.6 [6–224], 39.9 [7–160]	NR	NR	Exercise	Outpatient Rehabilitation
Nayak et al. (2025), India [29]	42, 42	57.6 ± 9.6, 59.3 ± 26.8	14.3, 28.6	74.4 ± 60.8, 80.4 ± 96.9	57.1, 69.0	NR	Combined	Community Health Centres/Home
Ramage et al. (2026), Australia [70]	20, 20	59 ± 16	40	39 [7–63]	NR	NIHSS: mild 100%	Combined	Home (Telehealth)
Siverstsen et al. (2022), Norway [57]	25, 30	73.0 ± 10.4, 69.3 ± 10.6	52.0, 23.3	NR	96.0, 86.7	NIHSS: 5.0 ± 1.1, 3.6 ± 0.6	Exercise	Inpatient and Outpatient Rehabilitation
Swank et al. (2020), United States [61]	37, 36	61.2 ± 16.9, 61.3 ± 15.2	51.2, 41.7	NR	NR	NIHSS: 5.04 ± 1.08, 3.64 ± 0.58	Exercise	Inpatient Rehabilitation
Telfils et al. (2023), France [58]	41, 42	62.2 ± 13.6, 62.2 ± 13.6	32.5, 32.5	2.6 ± 1.5, 2.6 ± 1.5	75, 75	Both-MI: 94 [77–100]	BCT	Home
Thompson et al. (2024), United States [14] *	81/80, 89	62.0 ± 1.4/ 62.0 ± 1.5, 63.0 ± 1.3	48.2/45.0, 46.1	46 ± 8.1/ 42 ± 5.5, 46 ± 6.6	NR	NR	BCT/Combined/Exercise	Laboratory
Vanroy et al. (2017), Belgium [62]	33, 26	66.7 ± 8.8, 63.8 ± 11.8	39.4, 30.8	1.7 ± 0.7, 1.6 ± 0.6	87.9, 84.6	NIHSS: 5 [3–7], 5 [2–10]	Combined	Inpatient Rehabilitation
Waddell et al. (2022), United States [67]	17, 17	57 ± 13.8, 61 ± 16.9	64.7, 64.7	16.2 ± 9.2, 39.2 ± 82.1	64.7, 70.6	NR	BCT	Home
Wright et al. (2021), United Kingdom [68]	16, 18	59.6 ± 10.1, 65.1 ± 10.1	12.5, 22.2	13 ± 19, 32 ± 21	94.0, 78.0	mRS: 3.3 ± 0.6, 3.3 ± 0.7	Exercise	Home

Values are presented as mean ± standard deviation, median [interquartile range or range], n or %. Abbreviations: I, intervention; C, control; NR, not reported; NIHSS, National Institutes of Health Stroke Scale; mRS, modified Rankin Scale; MAS, Modified Ashworth Scale; FMA, Fugl-Meyer Assessment; FAC, Functional Ambulation Category; SSS, Scandinavian Stroke Scale; MI, Motoricity Index. * Asterisked studies have 3 arms. Intervention type classified as Exercise-only, behaviour change technique (BCT)-only, or combined. Setting reflects the primary environment in which the intervention was delivered.

**Table 2:** Intervention characteristics and activity monitoring.

Study	Type	Intervention	Frequency	Intensity	Time	Setting	Monitor (Placement)
Alvarenga [69]	BCT	Sociocognitive theory + goal setting	2×/week first month, 1×/week for the other 2 months	As tolerated	60 min; 3 months	Home	Actigraph wGT3X-BT (waist), Xiaomi Corporation, Ltd. consumer smart band (wrist)
Ashizawa [43]	BCT	SB reduction + goals	2×/week (inpatient)	NR	20 min; 3 months	Hospital/home	Active Style Pro (waist)
Ashizawa [44]	BCT	SB reduction + monitoring	2×/week (inpatient)	NR	20 min; 3 months	Hospital/home	Active Style Pro (waist)
Brauer [45]	Combined	Treadmill + self-management	3×/week	40–60% HRR	30 min; 8 weeks	Rehab	ActivPAL (thigh)
Chiong [59]	Exercise	Stretching + orthotic	Daily	NR	24 weeks	Outpatient	Pedometer (NR)
Danks [46]	Combined	Treadmill + step feedback	3×/week	~80% HRR	40 min; 12 weeks	Laboratory	StepWatch (ankle)
Dean [60]	Exercise	Task-oriented training	≥3×/week	NR	45–60 min; 40 weeks	Home/centre	Pedometer (hip)
De Rooij [56]	Exercise	Gait training	2×/week	NR	30 min; 6 weeks	Rehab	DynaPort (waist)
Dorsch [47]	BCT	Feedback (activity graphs)	3×/week	NR	NR	Inpatient rehab	Gulf Coast (ankle)
Duncan [48]	Exercise	BWSTT + overground	3×/week	Moderate	~80 min; 12–16 weeks	Inpatient/home	SAM (ankle)
English [49]	BCT	Motivational interviewing	4 sessions (total)	NR	NR	Home	ActivPAL/Actigraph (thigh/waist)
Givon [65]	Exercise	Gaming-based exercise	2×/week	NR	60 min; 12 weeks	Rehab	Actical (hip)
Hornby [63]	Exercise	High-intensity walking	3–5×/week	70–80% HRmax	60 min; 8 weeks	Lab/outpatient	SAM (ankle)
Ivey [64]	Exercise	Treadmill training	NR	50–80% HRR	50 min; 6 months	Lab/centre	SAM (ankle)
Kanai [50]	Combined	Rehab + feedback	5–6×/week	40–60% HRmax	40–120 min	Hospital	Fitbit (ankle)
Klassen [51]	Exercise	Walking training	5×/week	≥40–60% HRR	1–2 h/day; 4 weeks	Inpatient rehab	Fitbit (ankle)
Kono [52]	Combined	Lifestyle counselling	NR	NR	30–40 min	Home	Lifecorder (waist)
Krawcyk [54]	Exercise	HIIT	5×/week	77–95% HRmax	15 min; 12 weeks	Home	AX3 (thigh)
Mandigout [53]	BCT	Coaching + PA promotion	1×/week (contact)	NR	12 months	Home	SenseWear (arm)
Mansfield [55]	Combined	Walking + feedback	5×/week	NR	60 min	Inpatient rehab	X6-2 (ankle)
Meester [66]	Exercise	Treadmill + cognition	2×/week	55–85% HRmax	30 min; 10 weeks	Community	SAM (ankle)
Mudge [22]	Exercise	Circuit training	3×/week	NR	50–60 min; 4 weeks	Rehab	SAM (ankle)
Nayak [29]	Combined	Behaviour change + multimodal physical exercise	Daily	RPE 4-5	30 mins	Community health outreach centres + home	ActiGraph (hip)
Ramage [70]	Combined	Physical activity programme + behaviour change (i-REBOUND)	2×/week	55% to 90% HRmaax	20 mins	Home (Telehealth)	Fitbit (wrist)
Sivertsen [57]	Exercise	Core strengthening	3–6×/week	NR	60 min; 12 weeks	Mixed	Actigraph (waist)
Swank [61]	Exercise	Task-based training	Daily	NR	30 min ×2; 4 weeks	Inpatient rehab	Actigraph (waist)
Telfils [58]	BCT	Coaching + monitoring	Daily + weekly contact	NR	6 months	Home	SenseWear (arm)
Thompson [14]	Combined	Walking + SAM	3×/week	70–80% HRR	40 min; 12 weeks	Lab	Fitbit (ankle)
Vanroy [62]	Combined	Aerobic + education	3×/week	60–75% HRR	30 min; 9 months	Inpatient rehab	Digiwalker (hip)
Waddell [67]	BCT	Gamification	Daily	NR	8 weeks	Home	Fitbit (wrist)
Wright [68]	Exercise	Gait training	Daily	NR	≥30 min; 10 weeks	Home	ActivPAL (thigh)

Abbreviations: BCT, behaviour change technique; BWSTT, body-weight-supported treadmill training; HIIT, high-intensity interval training; HRmax, maximum heart rate; RPE: Rate of perceived exertion; HRR, heart rate reserve; NR, not reported; PA, physical activity; SAM, Step Activity Monitor; SB, sedentary behaviour.

**Table 3:** Group-level effect sizes (standardized mean difference) and 95% confidence intervals for outcomes included in meta-analysis.

Study	Intervention	Control	SMD (95% CI)			
			Steps per Day	SSGS	FGS	6MWT
Alvarenga, 2025 [69]	BCT (self-efficacy, goal setting and usual care)	Education on general stroke and usual care	0.83 (0.02, 1.64)	0.39 (−0.39, 1.17)	NA	0.39(−0.39, 1.17)
Ashizawa, 2022 [43]	BCT (education on reducing SB)	Exercise (education on increasing PA)	0.57 (0.07, 1.08)	NA	NA	NA
Ashizawa, 2023 [44]	BCT (education on reducing SB)	Exercise (education on increasing PA)	0.40 (−0.02, 0.83)	NA	NA	NA
Brauer, 2022 [45]	BCT + Exercise	Usual gait training	0.38 (0.01, 0.74)	0.50 (0.13, 0.86)	0.62 (0.25, 0.99)	NA
Chiong, 2012 [59]	Exercise and orthotic device	Exercise (stretching)	−0.22 (−1.39, 0.95)	NA	NA	NA
Danks, 2016 [46]	BCT + Exercise	Exercise (aerobic)	−0.56 (−1.31, 0.19)	−0.19 (−0.83, 0.44)	−0.15 (−0.78, 0.48)	0.00 (−0.73, 0.73)
Dean, 2012 [60]	Exercise (designed to enhance mobility and PA)	Exercise (designed to enhance upper limb mobility and cognition)	0.30 (−0.02, 0.62)	NA	NA	NA
De Rooij, 2021 [56]	Exercise (virtual reality gait training)	Exercise (treadmill training)	NA	NA	NA	−0.17 (−0.71, 0.37)
Duncan, 2011a [48]	Exercise (locomotor training using body weight)	Exercise (home-based)	NA	0.38 (0.14, 0.63)	NA	0.40 (0.16, 0.65)
Duncan, 2011b [48]	Exercise	Exercise (home-based)	NA	0.38 (0.14, 0.62)	NA	0.36 (0.12, 0.60)
Givon, 2016 [65]	Exercise (whole body video game exercise)	Usual care	0.35 (−0.22, 0.92)	0.49 (−0.08, 1.06)	0.67 (0.09, 1.25)	NA
Hornby, 2022a [63]	Exercise (high-intensity training in variable context)	Exercise (low-intensity variable stepping training)	0.38 (−0.24, 1.00)	0.65 (0.02, 1.28)	0.73 (0.09, 1.36)	0.71 (0.07, 1.34)
Hornby, 2022b [63]	Exercise (high-intensity forward training with minimal variability)	Exercise (low-intensity variable stepping training)	0.34 (−0.28, 0.96)	0.72 (0.08, 1.35)	0.84 (0.20, 1.48)	0.66 (0.03, 1.29)
Kanai, 2018 [50]	Exercise + BCT	Supervised rehabilitation programme	1.12 (0.56, 1.68)	NA	NA	NA
Klassen, 2020a [51]	Exercise (DOSE 1–30 min moderate intensity ≥40% progressing to >60% HRR)	Usual care	NA	0.46 (−0.09, 1.01)	NA	0.47 (−0.09, 1.02)
Klassen, 2020b [51]	DOSE 2—higher intensity training (extra 1-h session in addition to DOSE1)	Usual care	NA	0.59 (0.03, 1.15)	NA	0.49 (−0.07, 1.04)
Kono, 2013 [52]	BCT + Exercise	Advise on lifestyle modification	0.82 (0.34, 1.30)	NA	NA	NA
Krawcyk, 2019 [54]	Exercise (high-intensity interval training)	Usual care	−0.25 (−0.74, 0.24)	NA	NA	NA
Mandigout, 2020 [53]	BCT	Usual care	NA	NA	NA	0.16 (−0.26, 0.59)
Mansfield, 2015 [55]	BCT + Exercise	Usual care	0.21 (−0.29, 0.72)	0.18 (−0.32, 0.68)	NA	NA
Meester, 2019 [66]	Exercise (treadmill training with simultaneous cognitive demand)	Exercise (treadmill training without cognitive demands)	0.23 (−0.32, 0.78)	NA	NA	NA
Mudge, 2009 [22]	Exercise (group-based supervised exercise class)	Group-based social and educational sessions	0.64 (0.12, 1.16)	NA	NA	0.77 (0.24, 1.29)
Nayak, 2025 [29]	Multimodal exercise + behavioural counselling	One educational session	0.11 (−0.31, 0.54)	−0.52 (−0.96, −0.09)	−0.58 (−1.01, −0.15)	−0.47 (−0.90, −0.04)
Ramage, 2026 [70]	Combined exercise and behaviour support	Telehealth health education and advice	0.54 (0.09, 0.98)	NA	NA	NA
Sivertsen, 2022 [57]	Exercise (I-CoreDIST intervention)	Usual care	−0.40 (−0.90, 0.11)	−0.39 (−0.89, 0.12)	−0.44 (−0.95, 0.06)	NA
Swank, 2020 [61]	Exercise (patient-directed)	Usual care	0.47 (0.01, 0.93)	NA	NA	NA
Telfils, 2023 [58]	BCT	Usual care	NA	NA	NA	0.26 (−0.17, 0.69)
Thompson, 2024a [14]	BCT	Exercise	0.35 (0.05, 0.65)	−0.18 (−0.48, 0.12)	NA	−0.28 (−0.58, 0.02)
Thompson, 2024b [14]	BCT + Exercise	Exercise	0.23 (−0.07, 0.53)	−0.07 (−0.37, 0.23)	NA	−0.12 (−0.42, 0.18)
Wright, 2021 [68]	Exercise + technology	Usual care	0.51 (−0.16, 1.18)	NA	NA	0.43 (−0.23, 1.10)

Abbreviations: 6MWT, six-minute walk test; BCT, behaviour change technique; CI, confidence interval; DOSE, Determining Optimal post-Stroke Exercise; FGS, fastest gait speed; HRR, heart rate reserve; NA, Not Applicable; PA, physical activity; SB, sedentary behaviour; SSGS, self-selected gait speed; SMD, standardized mean difference.

## Data Availability

The data supporting the findings of this study are available from the corresponding author upon request.

## Supplementary Materials

The following supporting information can be downloaded at: https://www.mdpi.com/article/10.3390/s26144332/s1, Table S1: PRISMA checklist; Table S2: Search strategies; Table S3: Data extraction form (template); Table S4: Behaviour change technique (BCT) coding of included interventions; Table S5: Evaluation of methodological quality using the PEDro Scale; Table S6: Summary of GRADE Certainty of Evidence [14,22,29,43,44,45,46,47,48,49,50,51,52,53,54,55,56,57,58,59,60,61,62,63,64,65,66,67,68,69,70].

## Abbreviations

BCT	Behaviour Change Technique
CI	Confidence Interval
I	Intervention Group
C	Control Group
FAC	Functional Ambulation Category
FMA	Fugl-Meyer Assessment
GRADE	Grading of Recommendations Assessment, Development and Evaluation
HRR	Heart Rate Reserve
HRmax	Maximum Heart Rate
MAS	Modified Ashworth Scale
MI	Motoricity Index
mRS	Modified Rankin Scale
NIHSS	National Institutes of Health Stroke Scale
NR	Not Reported
PA	Physical Activity
PEDro	Physiotherapy Evidence Database
RCT	Randomized Controlled Trial
SD	Standard Deviation
SMD	Standardized Mean Difference
SSS	Scandinavian Stroke Scale
TUG	Timed Up and Go
6MWT	Six-Minute Walk Test
