# Supplementary material for: Effectiveness of Physical Rehabilitation Interventions on Walking Capacity and Wearable Sensor—Derived Performance After Stroke: A Systematic Review and Meta-Analysis of Randomized Controlled Trials
Source: Sensors (Basel). 2026 Jul 8;26(14):4332. doi: 10.3390/s26144332 (PMC13416881; doi:10.3390/s26144332)
Supplement: Supplementary file 1 [file sensors-26-04332-s001.zip › Supplementary Table S2 - Search Strategies.pdf]

Supplementary Table S2. Search strategies

Database	Search String	Records Found
**MEDLINE (Ovid)**	(Fitbit* OR "Apple Watch*" OR Garmin OR accelerometer* OR pedometer* OR "smart watch*" OR wearable technolog* OR body sensor*) AND (stroke OR poststroke OR cerebrovascular disease OR cerebrovascular accident* OR ischemic attack*) AND (physical activity OR sedentary behaviour OR sleep OR gait OR walking OR steps OR quality of life) AND (randomi* OR placebo OR trial OR groups)	471
**Embase (Ovid)**	(Fitbit* OR "Apple Watch*" OR Garmin OR ActivPAL OR Actigraph* OR accelerometer* OR wearable technolog* OR body sensor*) AND (stroke OR cerebrovascular disease OR cerebrovascular accident* OR ischemic attack*) AND (physical activity OR gait OR walking OR sedentary lifestyle OR sleep OR quality of life) AND (randomi* OR placebo OR trial OR rct)	695
**CINAHL**	(Fitness trackers OR Fitbit* OR "Apple Watch*" OR accelerometer* OR wearable technolog*) AND (Stroke OR cerebrovascular disorders) AND (physical activity OR gait OR sedentary behaviour OR sleep OR quality of life) AND (randomi* OR clinical trial OR placebo)	215
**Scopus**	TITLE-ABS-KEY (fitbit* OR "Apple Watch*" OR accelerometer* OR wearable technolog* OR smart watch*) AND TITLE-ABS-KEY (stroke OR cerebrovascular disease OR ischemic attack*) AND TITLE-ABS-KEY (physical activity OR gait OR walking OR sedentary OR sleep OR quality of life) AND TITLE-ABS-KEY (randomi* OR placebo OR trial OR groups)	773

Search Dates: Searches executed May 2026; no date restrictions applied
Deduplication: Performed using Covidence, followed by manual review
Software: Screening was conducted using Covidence (Veritas Health Innovation, Melbourne, Australia)
