# Supplementary material for: Effectiveness of Physical Rehabilitation Interventions on Walking Capacity and Wearable Sensor—Derived Performance After Stroke: A Systematic Review and Meta-Analysis of Randomized Controlled Trials
Source: Sensors (Basel). 2026 Jul 8;26(14):4332. doi: 10.3390/s26144332 (PMC13416881; doi:10.3390/s26144332)
Supplement: Supplementary file 1 [file sensors-26-04332-s001.zip › Supplementary Table S3 - Data Extraction Form.pdf]

Supplementary Table S3. Data Extraction Form (Template)

Study Design

RCT Type (e.g., pilot, parallel):	
Setting (e.g., inpatient, community):	
Country:	
Funding Source:	

Participant Characteristics

Number Randomized (N):	
Number Analyzed (N):	
Mean Age (± SD):	
Sex (% Female):	
Time Since Stroke (months):	
Stroke Type (% ischemic):	
Stroke Severity (e.g., NIHSS):	
Inclusion Criteria:	
Exclusion Criteria:	
Attrition (%):	

Intervention (Experimental Group)

Intervention Type (Exercise, BCTs, Combined):	
Frequency:	
Intensity:	
Time:	
Type:	
Setting:	
Activity Monitor (type and placement):	
Duration (weeks/months):	
Intervention delivery	

Comparator

Type:	
Frequency:	
Intensity:	
Time:	
Type:	
Setting:	
Activity Monitor (type and placement):	
Duration (weeks/months):	
Intervention delivery (if applicable	

Outcomes

Performance (e.g., steps/day):	
Capacity (e.g., gait speed, 6MWT):	
Measurement Tools:	
Time points:	
Effect Estimates:	
Confidence Intervals:	

Adherence and Safety

Adherence (% sessions completed):	
Adverse Events:	

Author Contact Information

Corresponding Author Name:	
Email:	
Institution:	

Notes

Additional Notes or Comments:	
