# Supplementary material for: Effectiveness of Physical Rehabilitation Interventions on Walking Capacity and Wearable Sensor—Derived Performance After Stroke: A Systematic Review and Meta-Analysis of Randomized Controlled Trials
Source: Sensors (Basel). 2026 Jul 8;26(14):4332. doi: 10.3390/s26144332 (PMC13416881; doi:10.3390/s26144332)
Supplement: Supplementary file 1 [file sensors-26-04332-s001.zip › Supplementary Table S4 - BCT coding.pdf]

Supplementary Table S4. Behaviour Change Technique (BCT) Coding of Included Interventions

Study	Intervention Type	BCT Code	BCT Label	Description
Alvarenga [69]	BCT	1.1	Goal setting (behaviour)	Goal attainment scaling
		1.4	Action plan	structured step-by-step physical activity plans
		2.2	Feedback on behaviour	Feedback on their daily step counts
		2.3	Self-monitoring of behavior	Tracking daily step volumes
		3.1	Social support (practical)	Engaged family members and caregivers
		5.1	Information about health consequences	Education on stroke inactivity health risks
		13.1	Identification of self as role model	Self-management principles
Ashizawa [43]	BCT	1.1	Goal setting (behaviour)	Step and screen-time targets
		2.3	Self-monitoring of behaviour	Tracking steps and sedentary behaviour
		2.2	Feedback on behaviour	Weekly feedback
		4.1	Instructions on how to perform the behaviour	Education on reducing sedentary behaviour
Ashizawa [44]	BCT	1.1	Goal setting (behaviour)	Step and screen-time targets
		2.3	Self-monitoring of behaviour	Monitoring activity patterns
		2.2	Feedback on behaviour	Weekly feedback

		4.1	Instruction on behaviour	Education sessions
Brauer [45]	Combined	1.1	Goal setting (behaviour)	Activity targets
		2.3	Self-monitoring of behaviour	Step tracking
		2.2	Feedback on behaviour	Progress feedback
		1.4	Action planning	Structured planning
		4.1	Instruction on behaviour	Education sessions
Danks [46]	Combined	1.1	Goal setting (behaviour)	Step progression targets
		2.3	Self-monitoring of behaviour	Step tracking
		2.2	Feedback on behaviour	Performance feedback
Dorsch [47]	BCT	2.2	Feedback on behaviour	Feedback via activity graphs
		2.7	Feedback on outcomes of behaviour	Feedback on walking performance
English [49]	BCT	1.1	Goal setting (behaviour)	Counselling to reduce sitting time
		4.1	Instruction on behaviour	Motivational interviewing
		3.1	Social support (unspecified)	Counselling sessions
Kanai [50]	Combined	1.1	Goal setting (behaviour)	Activity targets
		2.3	Self-monitoring of behaviour	Activity monitoring
		2.2	Feedback on behaviour	Accelerometer feedback
Kono [52]	Combined	4.1	Instruction on behaviour	Lifestyle education
		1.1	Goal setting (behaviour)	Activity goals
Mansfield [55]	Combined	2.2	Feedback on behaviour	Walking activity reports
		1.1	Goal setting (behaviour)	Step targets
		2.3	Self-monitoring of behaviour	Daily monitoring
Nayak [29]	Combined	1.1	BCT 1.1: Goal setting (behaviour)	Choosing daily physical activities

		1.4	Action planning	Scheduling daily physical activities
		2.2	Feedback on behaviour	Reviewing participant activity progress
		2.3	Self-monitoring of behavior	Recording daily logbook entries
		3.1	Social support (unspecified)	Caregivers encourage activities.
		4.1	Instructions on how to perform the behaviour	Teaching specific aerobic exercises
		5.1	Information about health consequences	Educating on stroke recovery
		7.1	Prompts/cues	Sending daily phone reminders.
		9.1	Credible source	Physiotherapists delivering structured sessions
		10.4	Social reward	Celebrating physical activity progress
Ramage [70]	Combined	1.1	Goal setting (behaviour)	Setting personal activity goals
		1.4	Action planning	Targeting specific exercise durations.
		2.3	Self-monitoring of behavior	Using self-report activity questionnaires.
		9.1	Credible source	Supervising exercises via telehealth
		4.1	Instructions on how to perform the behaviour	Instructing proper device attachment
Telfils [58]	BCT	1.1	Goal setting (behaviour)	Individualized PA goals
		2.3	Self-monitoring of behaviour	Wearable monitoring

		2.2	Feedback on behaviour	Weekly feedback
		3.1	Social support (unspecified)	Coaching calls and visits
		5.1	Information about health consequences	Education on PA
Thompson [14] (SAM arm)	BCT	1.1	Goal setting (behaviour)	Incremental step targets
		2.3	Self-monitoring of behaviour	Fitbit tracking
		2.2	Feedback on behaviour	Coaching feedback
		3.1	Social support (unspecified)	Behavioural coaching
Waddell [67]	BCT	1.1	Goal setting (behaviour)	Step goals (33–50% increase)
		2.2	Feedback on behaviour	Daily/weekly feedback
		10.4	Social reward	Gamification incentives
		3.1	Social support (unspecified)	Support partner involvement
Vanroy [62]	Combined	4.1	Instruction on behaviour	Education sessions
		3.1	Social support (unspecified)	Patient/family support

Note: BCT, Behaviour Change Technique; SAM, Step Activity Monitor
