# Supplementary material for: Effectiveness of Physical Rehabilitation Interventions on Walking Capacity and Wearable Sensor—Derived Performance After Stroke: A Systematic Review and Meta-Analysis of Randomized Controlled Trials
Source: Sensors (Basel). 2026 Jul 8;26(14):4332. doi: 10.3390/s26144332 (PMC13416881; doi:10.3390/s26144332)
Supplement: Supplementary file 1 [file sensors-26-04332-s001.zip › Supplementary Table S5 - PEDro.pdf]

Supplementary Table S5. Evaluation of methodological quality using the PEDro Scale

Study	1[*a]	2	3	4	5	6	7	8	9	10	11	Points
Alvarenga et al. [69]	Yes	Yes	Yes	Yes	No	No	Yes	Yes	Yes	Yes	Yes	8
Ashizawa et al. [43]	Yes	Yes	Yes	Yes	No	No	No	Yes	No	Yes	Yes	6
Ashizawa et al. [44]	Yes	Yes	Yes	Yes	No	No	Yes	Yes	Yes	Yes	Yes	7
Brauer et al. [45]	Yes	Yes	Yes	Yes	No	No	Yes	No	Yes	Yes	Yes	7
Chiong et al. [59]	Yes	Yes	Yes	Yes	No	No	Yes	Yes	No	Yes	Yes	7
Danks et al. [46]	Yes	Yes	Yes	Yes	No	No	Yes	Yes	No	Yes	Yes	7
Dean et al. [60]	Yes	Yes	Yes	Yes	No	No	Yes	Yes	Yes	Yes	Yes	8
De Rooij et al. [56]	Yes	Yes	Yes	Yes	No	No	Yes	Yes	Yes	Yes	Yes	8
Dorsch et al. [47]	Yes	Yes	Yes	Yes	No	No	Yes	Yes	No	Yes	Yes	7
Duncan et al. [48]	Yes	Yes	Yes	Yes	No	No	Yes	Yes	Yes	Yes	Yes	7
English et al. [49]	Yes	Yes	Yes	Yes	No	No	No	Yes	No	Yes	Yes	6
Givon et al. [65]	Yes	Yes	Yes	Yes	No	No	Yes	Yes	No	Yes	Yes	6
Hornby et al. [63]	Yes	Yes	Yes	Yes	No	No	No	No	No	Yes	Yes	5
Ivey et al. [64]	Yes	Yes	Yes	Yes	No	No	No	Yes	No	Yes	Yes	5
Kanai et al. [50]	Yes	Yes	Yes	Yes	No	No	Yes	Yes	No	Yes	Yes	7
Klassen et al. [51]	Yes	Yes	Yes	Yes	No	No	Yes	Yes	No	Yes	Yes	6
Kono et al. [52]	Yes	Yes	Yes	Yes	No	No	Yes	Yes	Yes	Yes	Yes	8
Krawcyk et al. [54]	Yes	Yes	Yes	Yes	No	No	No	No	Yes	Yes	Yes	5
Mandigout et al. [53]	Yes	Yes	Yes	Yes	No	No	No	Yes	Yes	Yes	Yes	7

Mansfield et al. [55]	Yes	Yes	Yes	Yes	No	No	Yes	Yes	No	Yes	Yes	7
Meester et al. [66]	Yes	Yes	Yes	Yes	No	No	Yes	Yes	No	Yes	Yes	6
Mudge et al. [22]	Yes	Yes	Yes	Yes	No	No	Yes	Yes	No	Yes	Yes	6
Nayak et al. [29]	Yes	Yes	Yes	Yes	No	No	Yes	Yes	Yes	Yes	Yes	8
Ramage et al. [70]	Yes	Yes	Yes	Yes	No	No	Yes	No	Yes	Yes	Yes	7
Sivertsen et al. [57]	Yes	Yes	Yes	Yes	No	No	No	Yes	Yes	Yes	Yes	7
Swank et al. [61]	Yes	Yes	Yes	Yes	No	No	Yes	Yes	No	Yes	Yes	7
Telfils et al. [58]	Yes	Yes	Yes	Yes	No	No	No	Yes	Yes	Yes	Yes	7
Thompson et al. [14]	Yes	Yes	Yes	Yes	No	No	Yes	Yes	Yes	Yes	Yes	8
Vanroy et al. [62]	Yes	Yes	Yes	Yes	No	No	Yes	Yes	Yes	Yes	Yes	8
Waddell et al. [67]	Yes	Yes	Yes	Yes	No	No	No	Yes	No	Yes	Yes	6
Wright et al. [68]	Yes	Yes	Yes	Yes	No	No	No	Yes	Yes	Yes	Yes	7

*a is excluded from the scoring
