# Supplementary material for: Effectiveness of Physical Rehabilitation Interventions on Walking Capacity and Wearable Sensor—Derived Performance After Stroke: A Systematic Review and Meta-Analysis of Randomized Controlled Trials
Source: Sensors (Basel). 2026 Jul 8;26(14):4332. doi: 10.3390/s26144332 (PMC13416881; doi:10.3390/s26144332)
Supplement: Supplementary file 1 [file sensors-26-04332-s001.zip › Supplementary Table S6- GRADE.pdf]

Supplementary Table S6: Summary of GRADE Certainty of Evidence

Outcome	Studies (n)	Participants	SMD (95% CI)	Certainty
**Exercise-only interventions**				
Steps/day	10	603	0.23 (0.02 to 0.44)	Moderate (⊕⊕⊕⊖)
Comfortable Gait Speed	5	648	0.36 (0.13 to 0.58)	Moderate (⊕⊕⊕⊖)
Fastest Gait Speed	3	165	0.40 (-0.22 to 1.02)	Very Low (⊕⊖⊖⊖)
Walking Endurance	6	685	0.41 (0.25 to 0.57)	Moderate (⊕⊕⊕⊖)
**Behaviour Change Technique (BCT)-only interventions**				
Steps/day	4	341	0.45 (0.22 to 0.69)	Moderate (⊕⊕⊕⊖)
Comfortable Gait Speed	2	194	-0.01 (-0.52 to 0.51)	Low (⊕⊕◯◯)
Walking Endurance (6MWT)	4	361	0.07 (-0.23 to 0.38)	Low (⊕⊕◯◯)
**Combined Exercise and BCT Interventions**				
Steps/day	8	664	0.38 (0.10 to 0.66)	Low (⊕⊕◯◯)
Comfortable Gait Speed	5	529	-0.01 (-0.37 to 0.35)	Very Low (⊕⊖⊖⊖)
Fastest Gait Speed	3	240	-0.02 (-0.74 to 0.70)	Very Low (⊕⊖⊖⊖)
Walking Endurance	3	280	-0.23 (-0.50 to 0.05)	Low (⊕⊕⊖⊖)
